# Stroke Mimic: A Case of Unilateral Thyrotoxic Hypokalemic Periodic Paralysis

**DOI:** 10.5811/cpcem.2019.11.44966

**Published:** 2020-01-24

**Authors:** Michael Lajeunesse, Scott Young

**Affiliations:** Madigan Army Medical Center, Department of Emergency Medicine, Tacoma, Washington

## Abstract

Thyrotoxic hypokalemic periodic paralysis (THPP) is a condition that results in transient skeletal muscle paralysis secondary to intracellular potassium sequestration. Susceptible individuals often have an underlying channelopathy, which may be exacerbated by lifestyle factors or underlying medical comorbidities such as hyperthyroidism or diarrheal illness. Classically, THPP presents with paralysis of proximal extremity musculature. In this case, we present a rare case of unilateral THPP. Such a presentation is relevant to emergency physicians as it mimics a stroke or transient ischemic attack and should be considered on the differential for unilateral neurologic deficits.

## INTRODUCTION

Thyrotoxic hypokalemic periodic paralysis (THPP) is an endocrinologic disease that presents with acute episodic paralysis. In THPP, a thyroid-hormone-mediated cascade results in increased sodium/potassium (Na^+^/K^+^) pump activity leading to K^+^ sequestration inside the cell and subsequent muscular paralysis.^1^,^2^ Underlying electrolyte channel mutations predispose individuals to acquired periodic paralysis syndromes, and the thyrotoxic insult instigates the episodic paralysis.^1^ THPP classically presents with proximal muscle weakness, with the lower extremities typically affected to a greater extent than the upper extremities.^1^ Atypical findings such as asymmetric paralysis are rare, and there is a paucity of such presentations reported in the literature. We report an atypical case of unilateral weakness secondary to THPP, adding this diagnosis to the long differential that emergency physicians must consider among other stroke mimics.

## CASE REPORT

A 48-year-old Caucasian male presented via emergency medical services (EMS) with sudden onset unilateral right-sided weakness. The patient reported a heavy sensation in his muscles on the right side of his body and complained of difficulty manipulating his phone. The peak symptoms lasted about 3–5 minutes, and by the time EMS arrived, gross motor function had returned. The patient reported persisting, mild, right-sided weakness on arrival to the emergency department (ED), but otherwise remained hemodynamically stable.

He reported a history of Grave’s disease controlled by methimazole and propranolol. Further history revealed a similar episode in December 2017 marked by generalized weakness and fasciculations. He had been found to have hyperthyroidism at that time and was started on propranolol, in addition to methimazole. His current medication list included methimazole, propranolol, telmisartan/hydrochlorothiazide, testosterone, albuterol, aspirin, and emtricitabine/tenofovir disoproxil. He also admitted to non-compliance with his high potassium diet and supplementation.

Physical examination demonstrated 4/5 strength in both the right upper and lower extremities, as compared with 5/5 strength on the left. Proximal and distal muscles were affected equally. The patient denied any sensory deficits. The EMS report described near-complete loss of right-sided motor function with rapid improvement en route to the hospital. Relevant laboratory testing demonstrated a K^+^ of 3.4 milliequivalents per liter (mEq/L) (reference range 3.5–5.1 mEq/L), normal blood glucose, a thyroid stimulating hormone (TSH) of 0.02 microinternational units per milliliter (uIU/ml) (reference range: 0.270–4.320 uIU/ml) and a free-thyroxine (T4) of 2.34 nanogram per deciliter (ng/dL) (reference range: 0.80–1.80 ng/dL). Computed tomography (CT) of the head with and without angiography demonstrated periventricular and subcortical white matter densities, which were unchanged from prior imaging, but did not reveal any intracranial hemorrhage or flow-limiting lesions. His electrocardiogram (ECG) showed a right bundle-branch block, which was unchanged from prior ECGs. Subsequent outpatient testing for syphilis was negative.

The diagnosis of THPP was made in consultation with neurology based on past medical history, lab abnormalities, and negative CT/CT angiography imaging. Given his near-complete recovery after several hours of observation and a near normal K^+^, we withheld K^+^ supplementation and propranolol in the ED. Endocrinology advised that he increase his methimazole and restart his high-potassium diet. The patient was discharged with endocrinology and neurology follow-up. Subsequent outpatient electroencephalogram performed by neurology in evaluation of atypical seizures was also negative. With compliance of his medications and high potassium diet, the patient has not had any additional episodes of paralysis.

## DISCUSSION

THPP has a 0.1–0.2% prevalence in the United States, typically presenting with transient symmetric skeletal muscle paralysis, which usually affects the lower extremities disproportionately to the upper extremities.^3^,^4^,^5^ It occurs more frequently in Asian populations and typically presents in Asian men ages 20 to 40.^3^ Males are more susceptible than females, with reported ratios ranging from 17:1 to 70:1.^6^ THPP is thought to be mediated through the Na^+^/K^+^ pump. Studies suggest that androgen-mediated promotion of the Na^+^/K^+^ pump and a larger muscle-to-body ratio lead to greater risk of THPP episodes.^1^ In addition, estrogen decreases Na^+^/K^+^ pump activity, making females less susceptible to this syndrome. Hyperthyroidism upregulates the Na^+^/K^+^ pump expression on skeletal muscle in addition to increasing the pump activity.^1^,^2^,^4^ As a result, the cell sequesters K^+^ intracellularly, leading to the common pathway for many acquired paralysis syndromes: hypokalemia.^6^

Thyrotoxicosis alone, however, does not result in episodic paralysis, nor do free T4 levels correlate to symptoms.^7^ Rather, certain genetic factors in combination with a trigger such as thyrotoxicosis lead to clinical manifestation of the disease.^8^

The potassium inwardly-rectifying channel subfamily J member 2 gene (KCNJ2) and potassium inwardly-rectifying channel subfamily J member 18 gene (KCNJ18) are the two most prominent genes with mutations that predispose individuals to THPP in Asians and Caucasians, respectively.^6^ These genes are inward- rectifying K^+^ channels, which counteract the Na^+^/K^+^ pump by channeling K^+^ extracellularly.^8^ In mutated genes, excess thyroid hormone, excess catecholamines or excess insulin may all inhibit efflux of potassium from the cell, further sequestering K^+^ and contributing to intravascular hypokalemia.^8^ Other known triggers include large carbohydrate loads (excess insulin), exercise (excess catecholamines), stress, toxic adenomas, diuretics, fluoroquinolones, aminoglycosides, amiodarone, alcohol, and even licorice.^2^,^8^,^9^,^10^

**CPC-EM Capsule**

What do we already know about this clinical entity?

Thyrotoxic hypokalemic periodic paralysis (THPP) is marked by transient hypokalemia due to hyperthyroidism resulting in transient symmetric muscular paralysis.

What makes this presentation of disease reportable?

THPP usually manifests as bilateral weakness. However, this case presented with unilateral weakness presenting as a stroke mimic.

What is the major learning point?

THPP is a stroke mimic and should be considered on the differential in patients with thyroid conditions.

How might this improve emergency medicine practice?

Physicians may now better identify this rare stroke mimic and initiate proper treatment while avoiding unnecessary testing.

Presentations vary widely from mild, transient, self-limited motor dysfunction to total flaccid paralysis including respiratory muscles.^11^,^12^,^13^ Bulbar and ocular symptoms have been reported in rare cases; however, it is unclear whether ocular involvement stems from THPP or the thyrotoxicosis.^1^,^8^ This syndrome may even precipitate various dysrhythmias including ventricular fibrillation, ventricular tachycardia, atrioventricular block, or sinus arrest.^14^

Although existence of asymmetric THPP is suggested in the literature, a paucity of unilateral or asymmetric cases has been reported.^1^,^15^ A small 2012 case series of 11 patients with acquired hypokalemic periodic paralysis reported asymmetric weakness in three patients; however, all of these patients had acquired non-thyrotoxic hypokalemic periodic paralysis, whereas no patients with THPP had asymmetric weakness.^15^ Treatment for THPP involves correction of K^+^ and returning the patient to a euthyroid state.^1^,^2^,^6^,^14^

Care must be taken when correcting K^+^ as the patient has relative hypokalemia, in which the potassium is merely shifted into the cell instead of being depleted in the body.^1^,^2^ In addition, similar to the presented patient who was borderline hypokalemic, one case of THPP with normokalaemia has been reported.^2^ This, however, may represent a redistribution of K+ back into the extra-cellular space at the time of the lab draw rather than true normokalemia at the time of symptom onset. Thus, potassium supplementation in the ED must be performed with care as 70–80% of patients treated with potassium have rebound hyperkalemia.^6^,^14^ Providers must also consider management of associated electrolytes such as magnesium when repleting potassium. Close cardiac monitoring is also essential since patients are at risk of both hypo and hyperkalemic-induced dysrhythmias.

In the ED, a non-selective β-blocker such as propranolol is favored to reduce hyperthyroid symptoms and inhibit intracellular potassium sequestration.^1^,^2^ Selective β-blockers do not act on skeletal muscle, which makes them less useful in the management of THPP.^1^ One study demonstrated resolution of paralysis and normalization of the potassium within two hours of a three milligram per kilogram dose of propranolol administration by mouth.^16^ After correction of the emergent episode, maintenance of a euthyroid state and outpatient follow-up is important in preventing recurrent paralytic episodes.^6^ This is critical for long-term management since repeated attacks may cause permanent muscle weakness.^1^

## CONCLUSION

In this case, we report a rare presentation of THPP associated with transient, self-resolving, unilateral weakness. While non-thyrotoxic hypokalemic periodic paralysis has been associated with asymmetric weakness, only one source cites THPP with asymmetry.^15^ Additional pathologies such as familial periodic paralysis, stroke, transient ischemic attack, and neurosyphilis were investigated both in the ED and in follow-up without evidence of another convincing etiology. Unilateral weakness in THPP is a rare stroke mimic. Nevertheless, it is an important diagnosis in the ED given that it requires prompt treatment, astute electrolyte management, and close cardiac monitoring, which sometimes may present a difference in priorities for stroke workups. TSH, rapid electrolyte testing, a history of hyperthyroidism, or a history of past similar episodes can help tip off a physician to this pathology.

For emergency physicians, ensuring proper follow-up is essential since compliance with medications and management of the patient’s hyperthyroidism can prevent long-term morbidity such as persistent muscular weakness. No studies have researched the propensity for repeated paralytic episodes or long-term morbidity in populations with disparate degrees of healthcare access; however, special attention should be paid to the disposition and follow-up of THPP patients with poor healthcare access who may not have easy access to medications or a physician to make medication changes. As emergency physicians, we can make a lasting difference for these patients by prompt recognition and proper care in the ED, and via coordination of appropriate outpatient management for medical comorbidities.
